# Supplementary material for: In Vivo Efficacy of SQ109 against Leishmania donovani, Trypanosoma spp. and Toxoplasma gondii and In Vitro Activity of SQ109 Metabolites
Source: Biomedicines. 2022 Mar 14;10(3):670. doi: 10.3390/biomedicines10030670 (PMC8944987; doi:10.3390/biomedicines10030670)
Supplement: Supplementary file 1 [file biomedicines-10-00670-s001.zip › biomedicines-1623562-supplementary.pdf]

Supplementary Figure S1

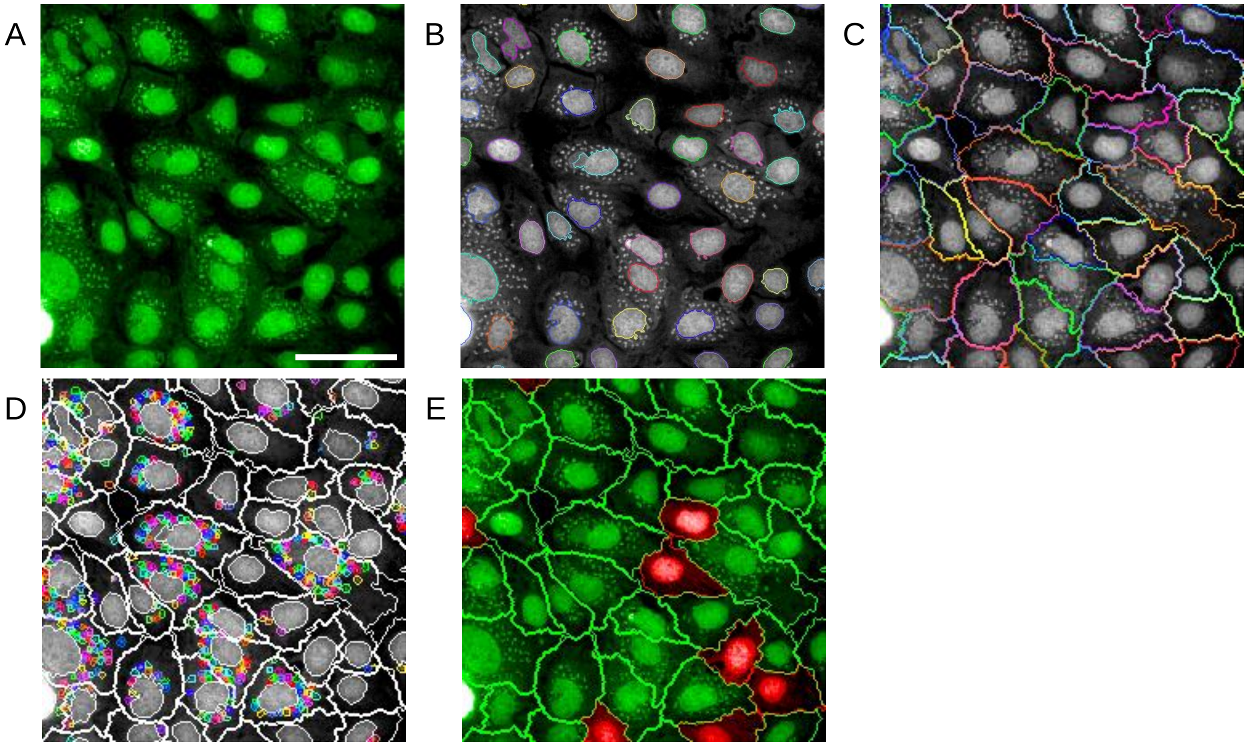

Supplementary Figure S1. Image analysis of intracellular *T.cruzi* by Columbus software. (A) Draq-5-stained image of U2OS infected with *T.cruzi* Dm28c at 3dpi. (B) Detection of large-sized nucleus of host cells using Draq-5 signal. (C) The host cell boundary masking performed using the low-intensity Draq-5 signals from cytosols. (D) Detection of small-sized nucleus of parasites by Draq-5 signals within the area of masked host cell. (E) Detection of infected host cells (in green) and non-infected host cells (in red). Scale bar = 50 μm.

Supplementary Figure S2

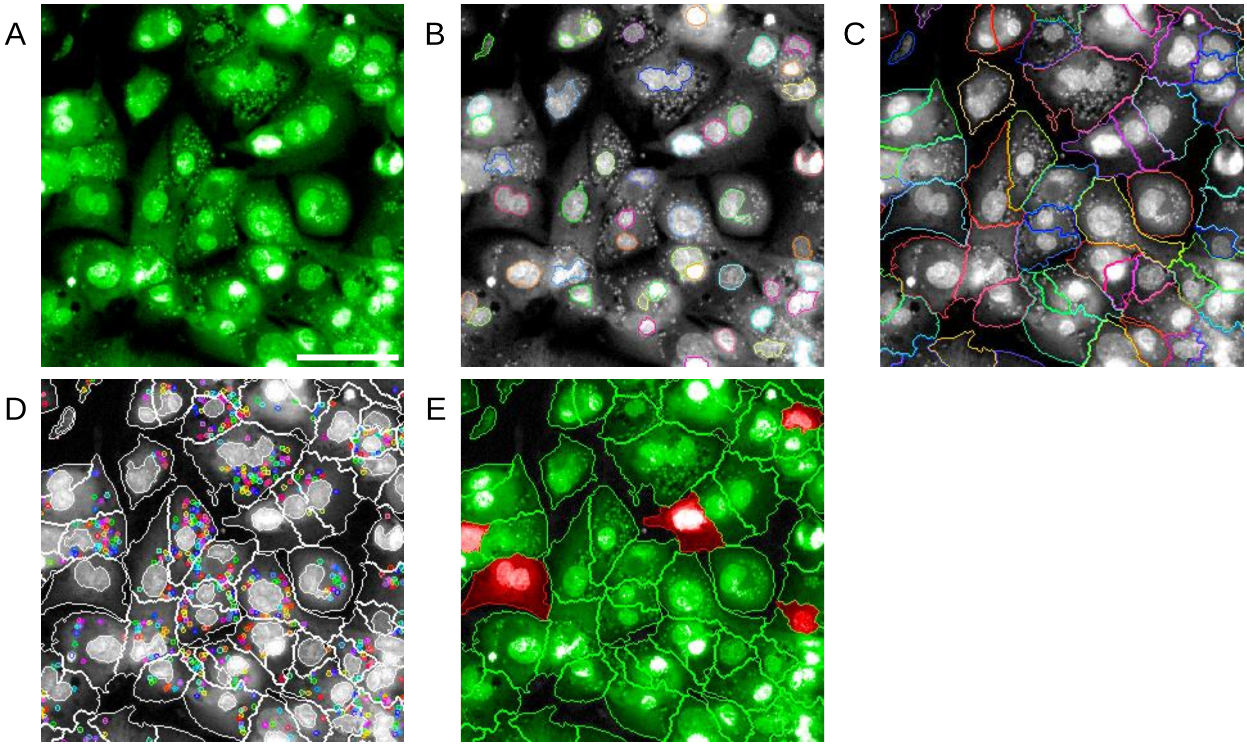

Supplementary Figure S2. Image analysis of intracellular *Leishmania* by Columbus software. (A) Draq-5-stained image of THP-1 infected with *L. donovani* amastigotes at 3dpi. (B) Detection of large-sized nucleus of host cells using Draq-5 signal. (C) The host cell boundary masking performed using the low-intensity Draq-5 signals from cytosols. (D) Detection of small-sized nucleus of parasites by Draq-5 signals within the area of masked host cell. (E) Detection of infected host cells (in green) and non-infected host cells (in red). Scale bar = 50 μm.

Supplementary Figure S3

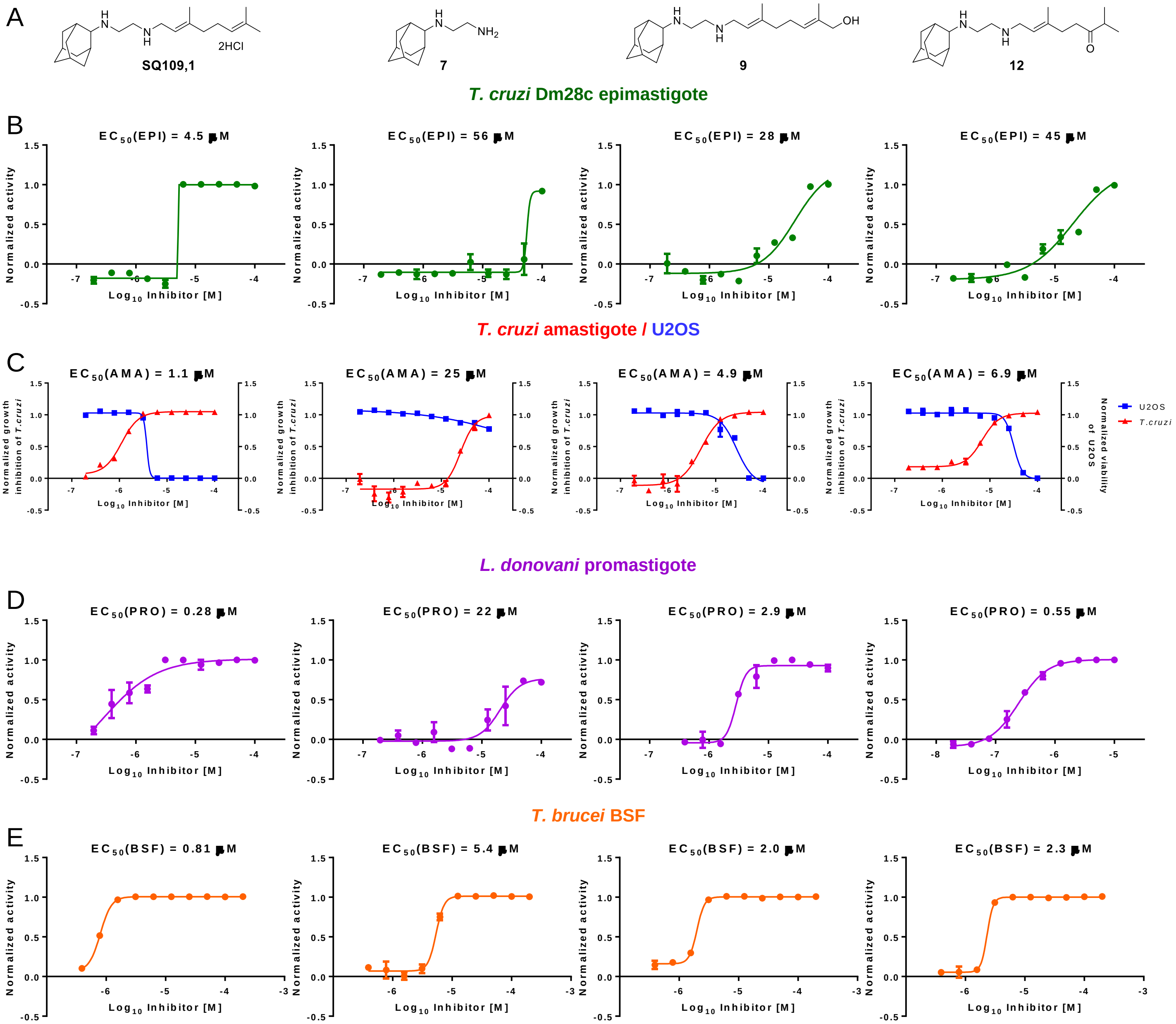

Supplementary Figure S3. Representative dose-response curves, for *T. cruzi* epimastigote, *T.cruzi* amastigote in U2OS, *L. donovani* promastigote and *T.brucei* BSF growth inhibition by SQ109, **7**, **9** and **12**. (A) Structures of compounds investigated. Dose-response curves for (B) *T. cruzi* Dm28c epimastigote (●), (C) *T.cruzi* amastigote (●) in U2OS (■), (D) *L. donovani* promastigote (●) and (E) *T.brucei* BSF (●) growth inhibition.

Supplementary Figure S4

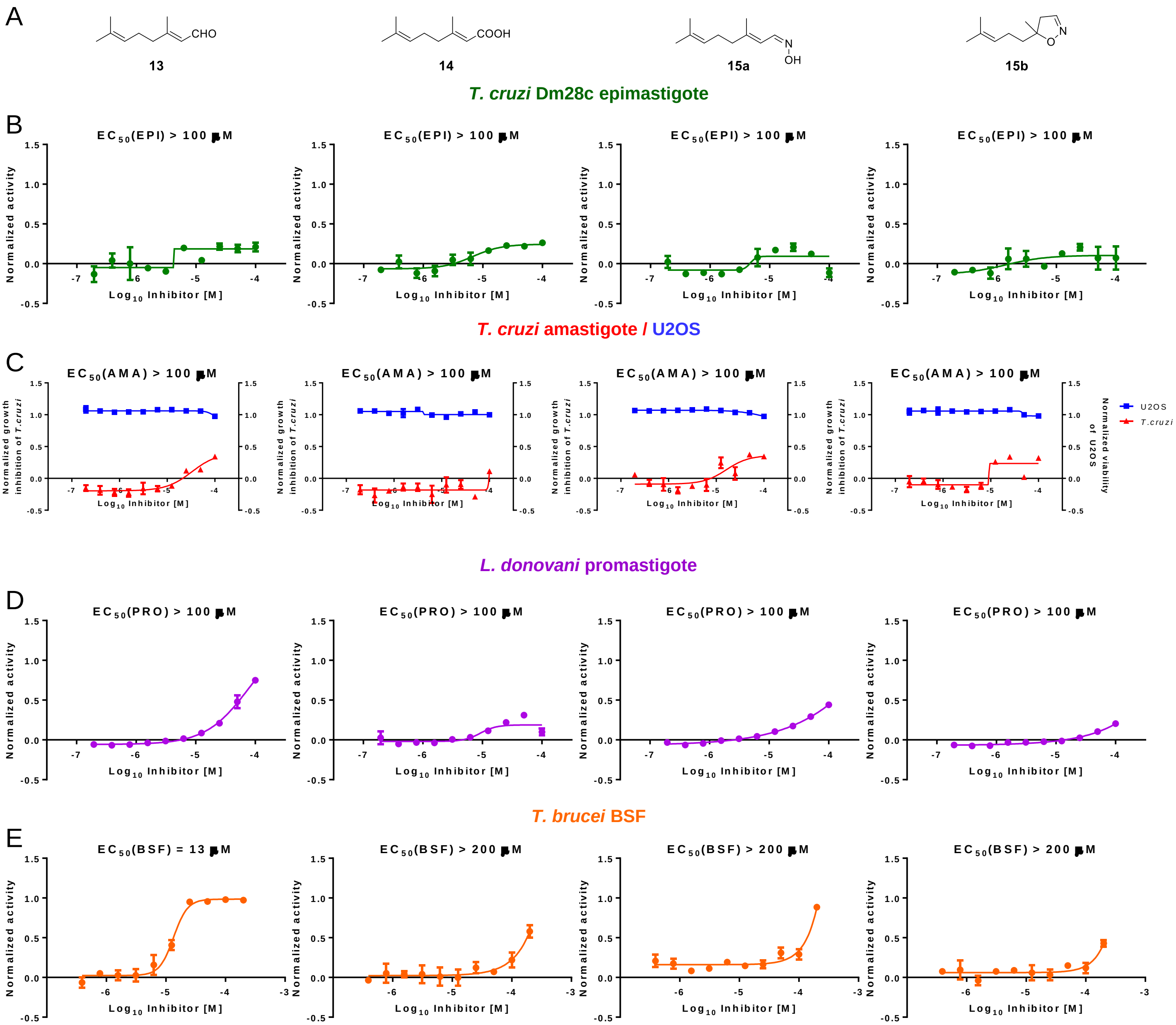

Supplementary Figure S4. Representative dose-response curves, for *T. cruzi* epimastigote, *T.cruzi* amastigote in U2OS, *L. donovani* promastigote and *T.brucei* BSF growth inhibition by **13**, **14**, **15a** and **15b**. (A) Structures of compounds investigated. Dose-response curves for (B) *T. cruzi* Dm28c epimastigote (●), (C) *T.cruzi* amastigote (●) in U2OS (■), (D) *L. donovani* promastigote (●) and (E) *T.brucei* BSF (●) growth inhibition.

Supplementary Figure S5

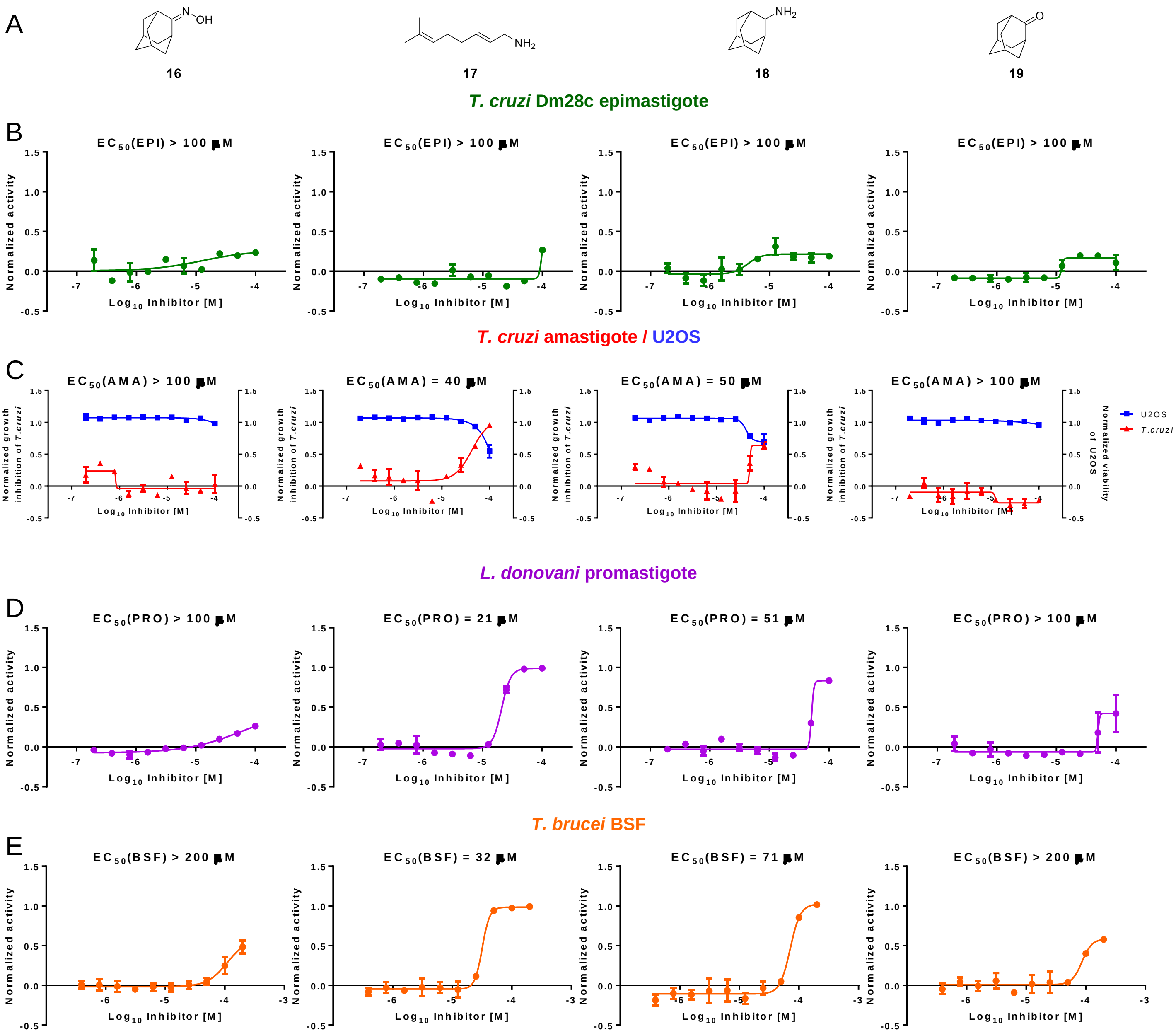

Supplementary Figure S5. Representative dose-response curves, for *T. cruzi* epimastigote, *T.cruzi* amastigote in U2OS, *L. donovani* promastigote and *T.brucei* BSF growth inhibition by **16**, **17**, **18** and **19**. (A) Structures of compounds investigated. Dose-response curves for (B) *T. cruzi* Dm28c epimastigote (●), (C) *T.cruzi* amastigote (●) in U2OS (■), (D) *L. donovani* promastigote (●) and (E) *T.brucei* BSF (●) growth inhibition.

Supplementary Figure S6

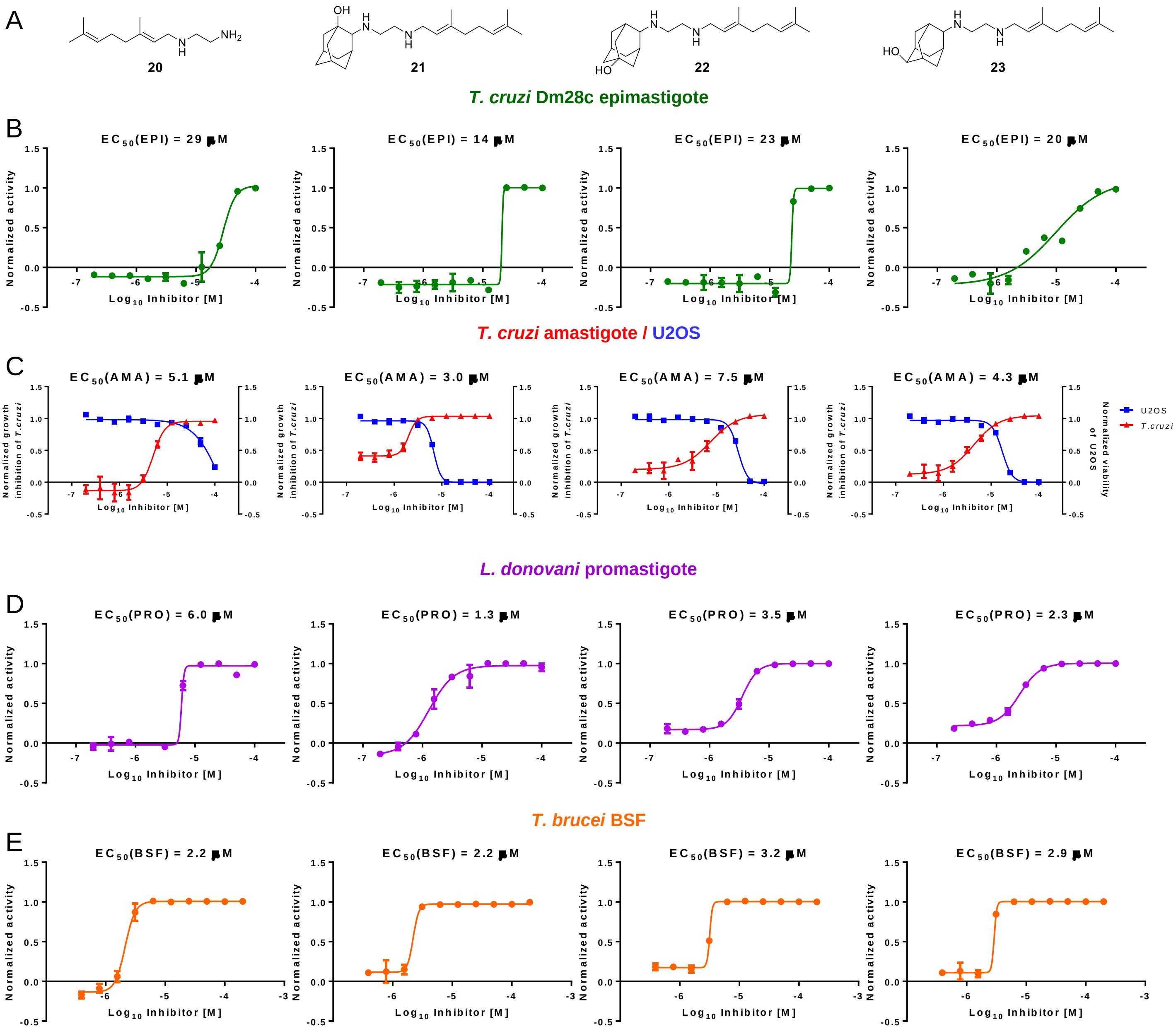

Supplementary Figure S6. Representative dose-response curves, for *T. cruzi* epimastigote, *T.cruzi* amastigote in U2OS, *L. donovani* promastigote and *T.brucei* BSF growth inhibition by **20**, **21**, **22** and **23**. (A) Structures of compounds investigated. Dose-response curves for (B) *T. cruzi* Dm28c epimastigote (●), (C) *T.cruzi* amastigote (●) in U2OS (■), (D) *L. donovani* promastigote (●) and (E) *T.brucei* BSF (●) growth inhibition.

Supplementary Figure S7

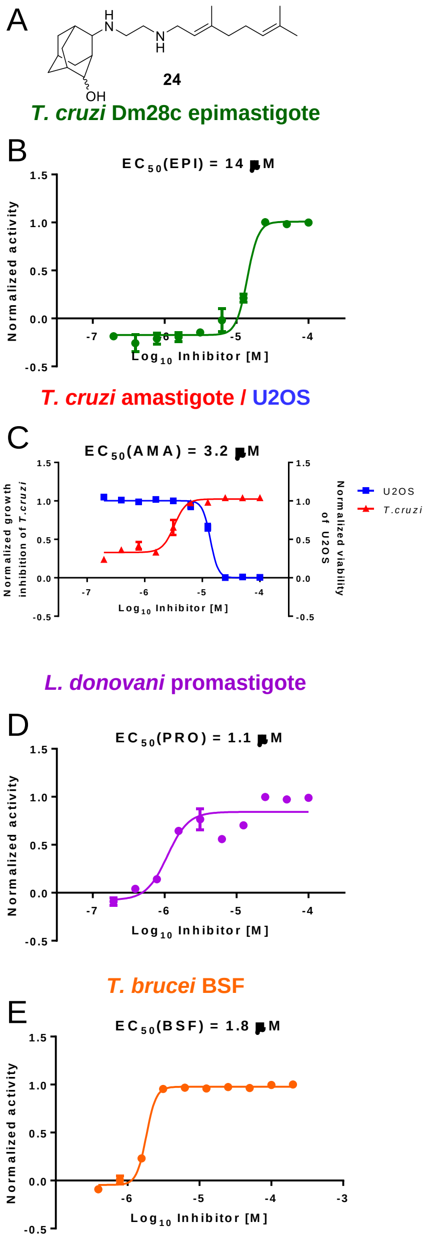

Supplementary Figure S7. Representative dose-response curves, for *T. cruzi* epimastigote, *T.cruzi* amastigote in U2OS, *L. donovani* promastigote and *T.brucei* BSF growth inhibition by **24**. (A) Structures of compounds investigated. Dose-response curves for (B) *T. cruzi* Dm28c epimastigote (●), (C) *T.cruzi* amastigote (●) in U2OS (■), (D) *L. donovani* promastigote (●) and (E) *T.brucei* BSF (●) growth inhibition.
